# Advantages and Limitations of Yellow Sticky Traps as a Pest Control Method in Pollinator-Friendly Rapeseed Agroecosystems: A Pilot Study in Northern Italy

**DOI:** 10.3390/insects17101035

**Published:** 2026-10-09

**Authors:** Francesco Lami, Olmo Scagliarini, Adele Bordoni, Lucia Lenzi, Serena Magagnoli, Marco Prandini, Giovanni Burgio, Fabio Sgolastra

**Affiliations:** 1 Department of Agricultural and Food Sciences (DISTAL), University of Bologna, Viale Giuseppe Fanin 42, 40127 Bologna, Italy; adele.bordoni@unifi.it (A.B.); lucia.lenzi4@unibo.it (L.L.); serena.magagnoli4@unibo.it (S.M.); giovanni.burgio@unibo.it (G.B.); fabio.sgolastra2@unibo.it (F.S.); 2 Centro Agricoltura Ambiente “Giorgio Nicoli”, Via Sant’Agata 835, 40014 Crevalcore, Italy; oscagliarini@caa.it (O.S.); mprandini@caa.it (M.P.); 3 Department of Biology, University of Florence, Via Madonna del Piano 6, 50019 Sesto Fiorentino, Italy

**Keywords:** pollination, integrated pest and pollinator management, mass-flowering crops, yellow sticky traps, *Brassicogethes aeneus*, *Ceutorhynchus obstrictus*, *Psylliodes chrysocephala*

## Abstract

Entomophilous mass-flowering crops such as rapeseed (*Brassica napus* L.) may especially benefit from Integrated Pest and Pollinator Management (IPPM) programs aimed at protecting the crop while reducing pollinator exposure to harmful pesticides. Yellow sticky trap barriers around field edges might represent an insecticide-free method of controlling immigrating pests. In this pilot study, we set up two experimental plots in a rapeseed agroecosystem in Northern Italy, comparing sticky traps with an insecticide treatment (acetamiprid), considering pest incidence/damage, pollinators/pollination services and yield. Sticky traps were associated with lower numbers of spring-immigrating pests (pollen beetles, weevils) and a lower percentage of seed pod damage. Overall yield was nonetheless higher in the insecticide treatment plot, possibly due to autumn-immigrating pest species which could be controlled by the insecticide but not by the sticky trap barrier (installed in spring). We found no significant effect of the treatments on pollinators or pollination, although plants treated with insecticide showed a higher average pollination deficit. Overall, our pilot study highlighted potential in yellow sticky traps as an IPPM measure, but they showed some limits—implying they would need to be paired with complementary methods, or to be installed earlier to intercept also autumn-immigrating pests.

## Simple Summary

Integrated Pest and Pollinator Management (IPPM) programs strive to protect crops while safeguarding pollinators from pesticides, which is especially important in mass-flowering crops such as rapeseed (*Brassica napus* L.). Yellow sticky trap barriers around field edges might represent an insecticide-free method of controlling immigrating pests; we tested this approach in comparison with an insecticide treatment (acetamiprid) in a rapeseed agroecosystem in Northern Italy, considering both pest incidence/damage and pollinators/pollination services. Sticky traps were associated with a more marked reduction in the populations of spring-immigrating pests such as pollen beetles and cabbage seed weevils, and of the related seed pod damage. Insecticide treatment, however, was nonetheless linked with a higher overall yield, possibly due to autumn-immigrating pest species which could be controlled by the insecticide but not by the sticky trap barrier (installed in spring). No significant effect was found for pollinators, albeit the insecticide treatment was associated with a higher average pollination deficit. Overall, our pilot study highlighted potential in yellow sticky traps as an IPPM measure, but they would need to be paired with complementary methods for more complete pest control, or to be installed earlier to intercept also autumn-immigrating pests.

## 1. Introduction

Rapeseed (*Brassica napus* L.) is one of the most important oil crops worldwide, accounting for roughly 50% of biofuel production in Europe [1]. As a mass-flowering crop, rapeseed can deeply influence pollinator communities in agricultural landscapes. Rapeseed provides a large but temporally concentrated pulse of floral resources, and its effects on pollinators may therefore depend on landscape composition, the availability of alternative floral resources, and the temporal scale considered. On the one hand, usually rapeseed flowering occurs over a relatively short period and may provide pollinators with resources only temporarily [2]. At high landscape-level cover, the resulting concentration of floral resources may also lead to transient pollinator dilution, as pollinators redistribute across the abundant rapeseed flowers [3,4]. On the other hand, when integrated appropriately within heterogeneous landscapes, these problems can be reduced [5,6] and rapeseed and other mass-flowering crops can provide substantial nutritional resources that support pollinator populations [7,8,9]. Such short-term increases in resource availability may improve pollinator survival and reproduction, potentially resulting in higher pollinator abundances in subsequent years through legacy effects [10]. Thus, the apparently contrasting effects of rapeseed may reflect differences in landscape context and temporal scale, with the same floral pulse potentially causing short-term redistribution of pollinators while contributing to longer-term population growth. In turn, pollination services provided by both wild and managed pollinators significantly increase rapeseed yield [11,12,13].

Entomophilous mass-flowering crops are ideal subjects for Integrated Pest and Pollinator Management (IPPM) programs, an expansion of Integrated Pest Management (IPM) with a heavy focus on pollinator conservation [14,15]. Even though rapeseed is self-compatible, with variability between cultivars and growing conditions [16], it is highly visited by insect pollinators, and its yield is usually significantly increased by them [17]. Such crops are the most likely to benefit from IPPM frameworks and, in turn, support large pollinator populations [18]. IPPM places a large emphasis on the ecology of both pollinators and pests [19,20] with the aim of identifying and deploying pollinator-friendly methods of pest control, thus maximizing both pollination and crop protection. The use of selective products, the attentive management of application timing and, most importantly, the overall reduction in pesticide use are all pivotal aspects of IPPM [14,21], as agrochemicals can often impact pollinators with negative consequences on pollination services—and thus on yield and product quality [22,23]. At the same time, the benefits of pollination may be reduced in plants stressed by excessive pest pressure [24,25], so efficient pest control methods alternative to pesticides are paramount in IPPM [19,26].

Sustainable and efficient pest control in rapeseed presents substantial challenges. Rapeseed is attacked by several species of beetles, flies, aphids and moths, with the highest pest diversity (17 species) being recorded in Europe [27,28]. Important pest species in these taxa include the common pollen beetle *Brassicogethes aeneus* (Fabricius), the brassica pod midge *Dasineura brassicae* (Winnertz), the cabbage aphid *Brevicoryne brassicae* (L.) and the diamondback moth *Plutella xylostella* (L.). Many of the most widely used neonicotinoid insecticides for the control of rapeseed pests (imidacloprid, thiamethoxam, clothianidin and thiacloprid) have been banned (or their approval has expired) in Europe due to their impacts on pollinators [29]—this led to some criticism, although the putative negative effects of this ban on yield have been disputed [30,31]. Acetamiprid is an example of a neonicotinoid still allowed in most of Europe, as it is considered relatively safe for bees when properly used [32], albeit higher-than-average environmental concentrations may cause adverse effects [33,34]; for instance, negative effects of acetamiprid have been recently reported in the solitary bee *Osmia cornuta* (Latreille) when co-exposed with an EBI fungicide [35]. Indeed, the EU regulatory framework concerning acetamiprid still explicitly requires consideration of risks to bees and other non-target arthropods [36]. Pyrethroid insecticides are commonly employed but, aside from the potential impacts on the environment, they are losing efficacy due to the evolution of resistance in the target arthropods [37]. As for pesticide-free protection strategies, breeding pest resistance into rapeseed has traditionally been challenging and has seen little progress so far [38]. Biological control can be similarly challenging, albeit conservation measures enhancing natural predator and parasitoid communities have enjoyed some success [39,40,41].

A frequently employed instrument in IPM and IPPM programs is represented by sticky traps. Sticky traps are mostly used to monitor pest population density in order to decide whether and when to intervene with treatments [42]. These tools, however, may also be employed as mass-trapping devices used to directly control pest populations, especially in enclosed environments such as greenhouses [43,44]. In open field conditions, sticky traps are used less frequently for direct pest control, but some partial successes have been achieved—although often aided by attractants other than colour, or in conjunction with complementary pest control methods [45,46,47]. Knowledge on spatial ecology and phenology of pests may be relevant for a more efficient placement of traps. For instance, it is known that many major pests of rapeseed enter the fields from the surrounding habitats during the spring months, and thus during this period they are amassed at the marginal areas of the fields themselves, only aggregating at the centre of the fields after flowering starts [48,49,50]. This suggests that placing sticky traps at field edges since early in the season might efficiently intercept immigrating pests, preventing them from colonizing the field—an insecticide-free form of “barrier treatment” [51]. A potential unintended effect to consider is non-target captures of beneficial arthropods by sticky traps: they are usually reported to be very low, but they may happen, with frequency depending on taxon and trap features [52,53].

In this pilot study, we aimed to test edge-placed yellow sticky traps as a mass-trapping method for IPPM rapeseed agroecosystems, also considering the effects on pollinators and pollination services in comparison with a positive control (insecticide-treated plot). Specifically, the goals were to (i) assess the effect of sticky traps and insecticide treatments on the abundance of major rapeseed pests and on pest damage incidence on seed pods, and (ii) investigate the impact of each pest control method on the abundance of flower visitors, pollinator deficit, pollinator contribution to yield—and on yield in general. We expected the sticky trap treatment to be associated with similar levels of pest control and yield, higher numbers of pollinators and more complete pollination services compared with the insecticide treatment. Our results might be useful in informing future research aimed at defining efficient IPPM strategies for rapeseed.

## 2. Materials and Methods

### 2.1. Experimental Area

The study took place in 2023 in a farm located in Crevalcore, under Bologna province, in the Emilia-Romagna Region of Northern Italy (44°45′25.44″ N; 11°11′47.91″ E). The test field intended for the study consisted of 1.82 ha sown with rapeseed of the KWS Gordon variety on 5 October 2022. The test field was carefully divided in half to form two experimental plots of 0.9 ha each. During the pre-flowering phase (22 March 2023) one plot (intended as a positive control) was treated with acetamiprid (Kestrel© by Nufarm Italia, Bologna, Italy—200 g/L of active molecule); the product was applied via air-assisted boom sprayer at the recommended application rate of 0.3 L/ha, with a spray volume of 400 L/ha, and only one application was performed in accordance with the integrated production guidelines of the Emilia Romagna Region. Plastic yellow sticky traps (model Horiver© by Koppert Italia, Bussolengo, Italy, 25 × 10 cm) were installed on posts around the perimeter of the other, insecticide-free plot, at a distance of 2 m from each other. Traps were first installed at the height of 50 cm, slightly higher than the field vegetation; later in the season, as rapeseed grew, a second series of traps was installed at the height of 1 m. Damaged or dirty traps were routinely replaced through the season.

### 2.2. Pest Surveys

We performed weekly surveys of insect pests in the sticky trap and insecticide plots from the end of March to the end of May (8 surveys in total). For these surveys, we focused on the main rapeseed pests that are targeted by yellow sticky traps, belonging to the orders Coleoptera, Diptera and Lepidoptera. Surveys in each plot consisted of the visual inspection of 50 rapeseed plants in 5 selected spots (10 plants per spot), recording pest species and number of individuals. This type of sampling design is widely employed and recommended for rapeseed pests [54,55]. Survey spots were squares of roughly 25 m^2^; in each plot, the 5 spots were selected to be at least 30 m from the nearest plot margin and from other survey spots. During the same surveys, in proximity to each survey spot, we also checked a trap post (5 trap posts per plot, 2 traps each, 20 traps per survey) and recorded the number and species of pests and beneficial arthropods (pollinators and biological control agents) captured by the traps. During the last survey (3 May 2023), we also collected 50 randomly selected seed pods per plot (from 50 different plants), which were checked for pest damage (including the presence of pest larvae) and classified as either “damaged” or “undamaged”.

### 2.3. Pollinator Surveys and Pollination Experiment

During the peak flowering month of rapeseed (April), pollinator surveys were performed weekly in the sticky trap and insecticide plots, for a total of 4 surveys. Surveys always took place between 10:00 AM and 12:00 PM during sunny, windless days. In each plot, an operator slowly walked across the entire plot area for 30 min, recording the number of flower visits by pollinators, considering as “flower visits” only instances in which the insect interacted with the rapeseed flower reproductive parts. Pollinators were identified on-field either to species or morphospecies level; the use of the morphospecies concept allowed us to have an idea of the taxonomic diversity at each plot/round combination without collecting/disturbing the animals, although we could not estimate overall taxonomic richness in the entire sampling campaign [56].

Within the two experimental plots, we defined a 9 × 6 m area at a distance of at least 30 m from the closest plot margin. In each area, we placed 12 plastic posts in a grid (3 rows comprising 4 posts each), so that posts would be at a distance of at least 3 m from each other. In the close proximity of each post (within a roughly 50 cm radius), we selected 3 rapeseed plants (72 plants in total). All plants were comparable in size and developmental stage. In each plant triplet, one plant was selected as the control (open pollination), one plant was subjected to pollinator exclusion, and one plant was subjected to supplementary pollination [22,57] (Figure S1). Both pollination treatments were only carried out on the first seven branches of the plants (counting from the tip). Pollinator exclusion was performed by enclosing the upper branches of the plant in a tulle bag (white polyester, 1 mm mesh size) from right before flowering (end of March). The tulle bag prevented insect access and only allowed wind-mediated and self-pollination; bags were periodically moved or replaced with larger bags to accommodate plant growth. Tulle bags and cages are a standard method in pollinator exclusion experiments [58], including those involving rapeseed [59]; a potential drawback to consider when interpreting the results is the fact that bags can alter the light availability or microclimatic conditions inside them, albeit literature generally reports that such mesh-based exclusion structures usually cause relatively low alterations [60,61]. Supplementary pollination was performed weekly during the peak flowering month (April) by collecting flowers from nearby plants and rubbing their anthers on the stigmas of the flowers of experimental plants. We used the same procedure (i.e., one collected flower was used to pollinate 3 experimental flowers) consistently among plants and sampling dates.

At harvest (19 June 2023), the seed produced by the first seven branches (counting from the tip) of each experimental plant was collected and weighed as a measure of plant-level yield. Flower-level yield was calculated by dividing plant-level yield by the number of flowers counted on the considered branches. We also characterized overall yield (g/m^2^) in the sticky trap and insecticide plots by collecting all plants in four randomly placed 1 × 1 m areas in each plot, and weighing all seeds produced within these areas.

### 2.4. Data Analysis

The effect of pest control treatment on the total number of pest insects was tested using a Generalized Linear Mixed-Effects Model (GLMM) with a Poisson distribution (Model 1); spot ID (i.e., the identity of the survey spots in which rapeseed plants were sampled in groups of 10) was used as a random factor. The effect of pest control treatment on the percentage of seed pods damaged by pests was checked using a Generalized Linear Model (GLM) with a binomial distribution (Model 2).

For each experimental plant triplet, we calculated the pollination deficit (*PD*) according to the formula *PD* = (*S* − *O*)/*S*, where *S* is the yield of the supplementary pollination plant and *O* is the yield of the open pollination (control) plant; in cases where *O* was higher than *S*, *O* was used as the denominator [62]. Pollination deficit was calculated at both plant level and flower level (i.e., average yield per flower). Due to the lower number of samples (8–24), we used Wilcoxon rank-sum tests to assess the effect of the pest control strategy (sticky traps v. insecticide) on pollinator abundance and species richness, yield (g/m^2^) and pollination deficit at the plant and flower level.

We also employed Gaussian GLMMs to test the effect of pest control treatment, pollination treatment (open v. exclusion v. supplementary) and their interaction on plant-level and flower-level yield (Model 3–4). Post ID (i.e., in-field plant triplet position) was included as a random factor. Plant-level yield was square-root transformed and flower-level yield was log-transformed in order to better meet model assumptions. In case of significant differences, post hoc tests with Tukey adjustment were carried out to compare the treatments.

Analyses and visualizations were performed using packages stats, lme4, DHARMa, emmeans and visreg in R v4.4.1 [63,64,65,66,67]. Due to limitations in the scale of the study (see Section 4.4 for more details), all statistical analyses should be regarded as exploratory.

## 3. Results

### 3.1. Pest Incidence

During plant surveys, we recorded a total of 939 pest insects, the majority of which (690 individuals, 73% of the sample) were found in the insecticide plot. The most abundant species recorded during these surveys was the common pollen beetle *B. aeneus* (446 individuals, 47% of the sample), followed by 3 species belonging to the weevil genus *Ceutorhynchus* Germar, all of which were more abundant in the insecticide plot than in the sticky trap plot (Figure S2). We also counted a total of 1915 pest insects captured by the surveyed yellow sticky traps, with the most frequently captured taxa being *B. aeneus* (581 individuals, 30% of the sample), genus *Phyllotreta* Chevrolat (565 individuals, 30% of the sample) and *Ceutorhynchus pallidactylus* (Marsham) (523 individuals, 27% of the sample). The number of beneficial arthropods captured in sticky traps was low, with 27 ladybird beetles (Coleoptera: Coccinellidae), 15 hoverflies (Diptera: Syrphidae) and 5 lacewings (Neuroptera: Chrysopidae).

**Figure 1:**
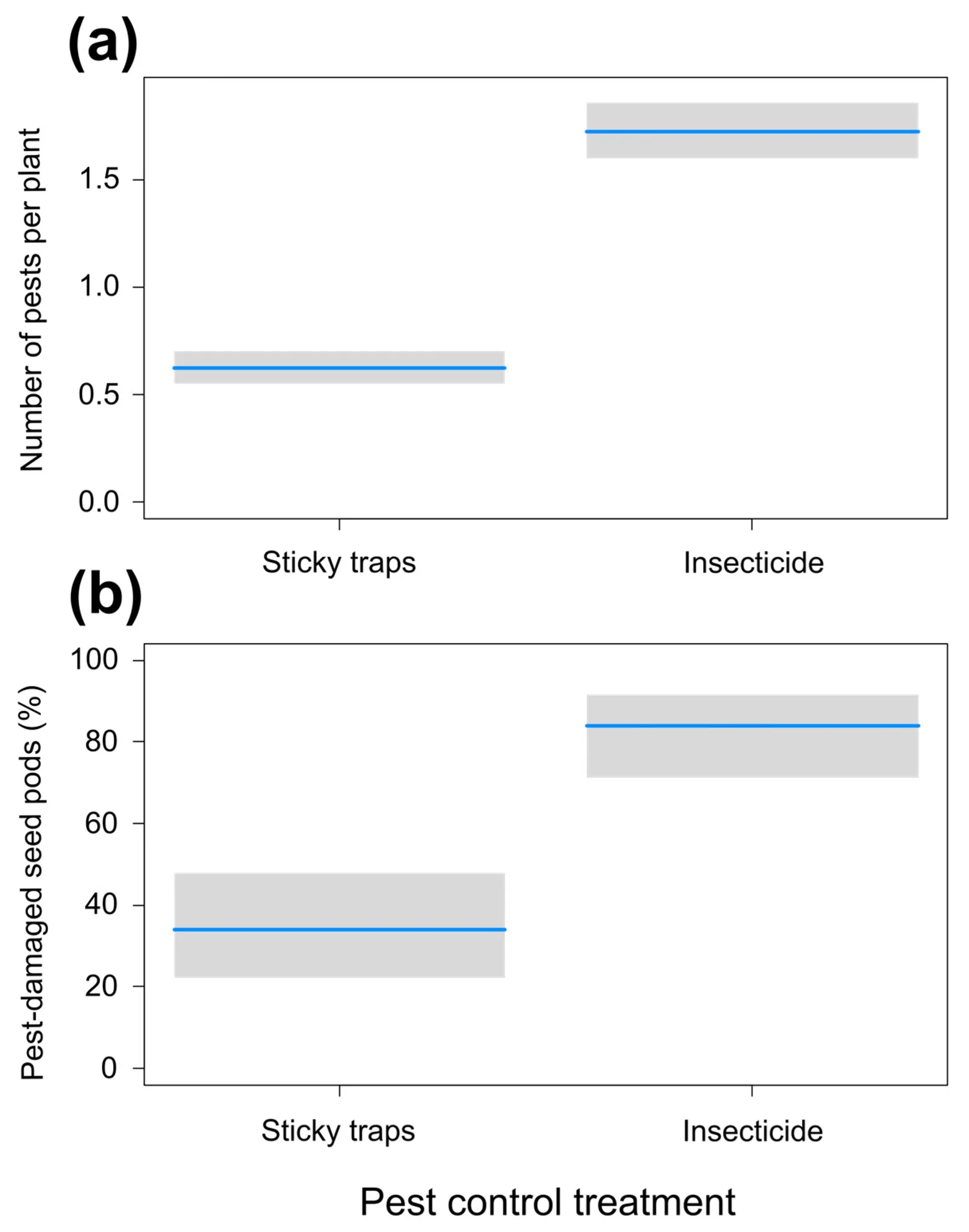
Effect of pest control treatment (yellow sticky traps v. insecticide) on the total number of pest insects recorded on rapeseed plants (**a**) and on the incidence of pest-related damage on seed pods (**b**), as a result of the exploratory statistical analysis. The fitted line indicates model predictions, while grey bands represent 95% confidence intervals.

The statistical models showed that the insecticide plot harbored a significantly higher number of the target pests (Table 1, Figure 1a) and had a significantly higher incidence of pest-damaged seed pods (Table 1, Figure 1b) than the sticky trap plot with yellow sticky traps.

### 3.2. Pollinators, Pollination and Yield

During our surveys, we recorded a total of 3034 pollinators visiting rapeseed flowers, the vast majority of them belonging to the western honeybee *Apis mellifera* L. (2852 individuals, 94% of the sample; Figure S3a). Among wild pollinators, the most abundant group was represented by hoverflies (Diptera: Syrphidae) with 60 individuals (2% of the sample; Figure S3b). According to the Wilcoxon rank-sum test, there were no significant differences between the sticky trap plot and the insecticide plot in terms of overall flower visitor abundance (*p*-value = 0.89) or species/morphospecies richness (*p*-value = 0.66).

**Figure 2:**
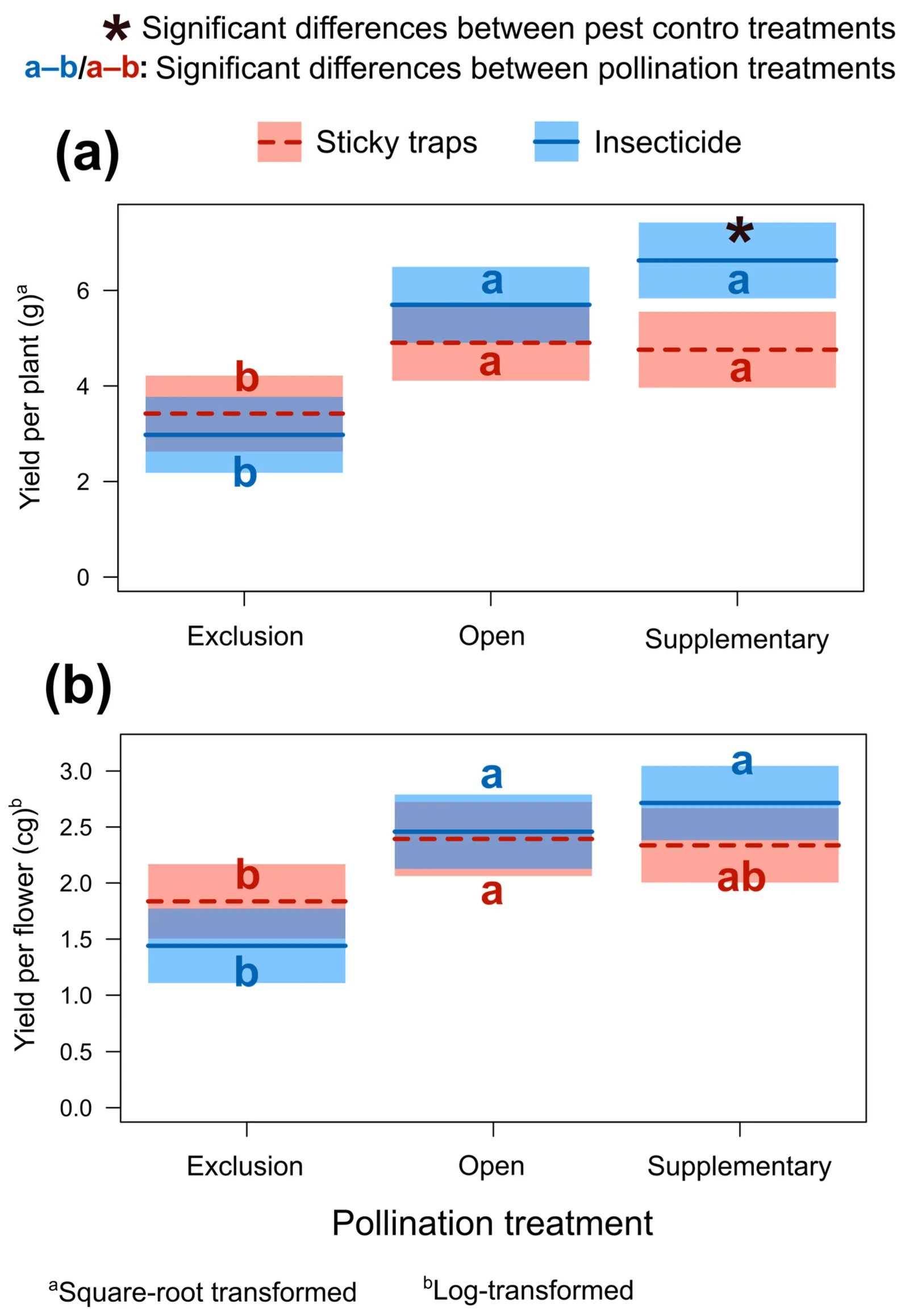
Interactive effect of pest control treatment and pollination treatment on rapeseed yield per plant (**a**) and per flower (**b**), as a result of the exploratory statistical analysis. Fitted lines indicate model predictions, while colored bands represent 95% confidence intervals. Different letters indicate statistically significant differences among pollination treatments for the sticky trap plot (in red) and for the insecticide plot (in blue), while asterisks indicate statistically significant differences between pest control treatments within the same pollination treatment, all according to post-hoc pairwise comparisons with Tukey adjustment.

There were significant interactions between pest control treatment and pollination treatment associated with changes in both plant-level and flower-level yield (Table 1, Figure 2a,b). Yield was significantly lower in the pollinator exclusion treatment in both pest control treatments, while there were no differences in yield between open pollination and supplementary pollination (Figure 2a,b). The difference between the insecticide plot and the sticky trap plot was more marked for the supplementary pollination treatment than for the open pollination treatment, with the insecticide plot being linked with a higher average yield; in particular, the insecticide plot was associated with a significantly higher plant-level yield than the sticky trap plot under supplementary pollination (Figure 2a). While the average pollination deficit in the insecticide plot (21% and 16% for plant-level and flower-level yield) was higher than the slightly negative pollination deficit in the sticky trap plot (−6% and −4% for plant-level and flower-level yield), variability was very high (with standard deviations of 42% and 48% for the insecticide plot, and 62% and 52% for the sticky trap plot, respectively), and the Wilcoxon rank-sum test did not detect significant differences between pest control treatments in this regard (*p*-values of 0.24 and 0.27 for plant-level and flower-level yield).

According to the Wilcoxon rank-sum test, the insecticide plot was associated with a significantly higher yield per m^2^ than the sticky trap plot (*p*-value = 0.03; Figure S4).

## 4. Discussion

Our study highlighted some potential advantages and limitations of yellow sticky traps as a pest control method for pollinator-friendly (IPPM) rapeseed agroecosystems, which could inform future research with important practical applications.

### 4.1. Seed Pod Pests and Damage

Compared with the insecticide treatment, yellow sticky traps were associated with a more marked reduction in the overall abundance of pollen beetles and pest weevils in rapeseed, with these arthropods also confirmed as among the most frequently caught species in the traps themselves. Both the pollen beetle and the *Ceutorhynchus* weevils are known to invade fields in early spring, initially aggregating at field edges [48,49,50]; they would therefore be indeed susceptible to traps placed at edges before field immigration starts. More importantly, the reduction in pest numbers seemingly had observable beneficial effects on damage control, as the percentage of damaged seed pods was significantly lower in the sticky traps plot than in the insecticide plot. Seed pod damage is usually associated with the cabbage seed weevil *Ceutorhynchus obstrictus* (Marsham) [28], which was indeed one of the most abundant pests in the insecticide plot, and which—like other pest species—was much less abundant in the sticky traps plot. Aside from direct damage, *C. obstrictus* activity is known to facilitate infestation by the brassica pod midge *D. brassicae* [68]; successful control of the weevil is therefore likely associated with a reduction in midge-related damage. If confirmed by further studies, these encouraging results would frame yellow sticky traps as a more efficient, pesticide-free alternative to the tested insecticide (acetamiprid) when considering protection from pollen beetles and weevils. Additionally, based on the extremely limited number of non-target captures compared with pest captures, it would be reasonable to assume that yellow sticky traps represent a negligible risk to beneficial arthropods, in accordance with available literature [52,53]; as we had no way to compare non-target mortality in the two systems, however, this topic should also be further investigated in future studies.

### 4.2. Pollinators

No difference was observed between the two treatments in terms of the abundance of pollinators. As it often happens in agroecosystems [69], the vast majority of flower visitors were honeybees, and our results are in line with available knowledge that, at recommended field doses, acetamiprid has a relatively low toxicity on this species [32,70]. This is also reflected by the pollination service, which was likely mostly provided by honeybees, given their dominance in the community. Consistent with that, there were no significant differences in terms of pollination deficit, and pollination deficit itself was negligible (no significant differences between open and supplementary pollination) in both the sticky traps plot and the insecticide plot. While pest control treatment did not affect pollinator taxonomic richness either, it is also difficult to draw even preliminary conclusions about effects on the abundance of wild flower visitors, as the latter were relatively scarce. Areas with lower densities of honeybees, which can compete with wild pollinators and exclude them from the most abundant flower resources [71,72,73], might be used to repeat the experiment and clarify the effects of treatments on more diverse pollinator communities. The effects of acetamiprid are in fact much less studied in wild pollinators than in honeybees, with some experiments highlighting negative effects at field-realistic doses [74,75].

### 4.3. Yield and Pollination

While yellow sticky traps were associated with a lower number of pollen beetles and weevils, some potential key limitations of the method (at least in the form applied here) were highlighted when considering yield-related measurements. Most importantly, in spite of the lower percentage of damaged seed pods, overall yield per m^2^ still managed to be lower in the sticky traps plot. This might suggest the presence of pests that, while undetected during the field samplings, were either more efficiently controlled by acetamiprid than by yellow sticky traps, or enjoyed competitive release from other pest species in the sticky trap plot [76]. This would also explain the higher average plant-level and flower-level yield in the insecticide plot under favorable pollination conditions (open/supplementary pollinations), with the difference being significant for plant-level yield under supplementary pollination; pest control and pollination often act synergistically, so increasing pest pressure may reduce pollination benefits [24,25]. The higher average pollination deficit in the insecticide plot might also be linked with this: plants less stressed by pests would potentially benefit more from pollination, so their pollination deficit is higher even when their overall yield is higher. However, differences in pollination deficit between treatment plots were non-significant (and variability very high), so this should be considered a hypothesis to be tested in future studies.

Aphids are important rapeseed pests worldwide through direct feeding and pathogen transmission [77], and are among the main targets of acetamiprid [78], so they may fit the observed patterns; however, almost no aphids were seen on plants in either plot during fieldwork. A more likely candidate is the cabbage stem flea beetle *Psylliodes chrysocephala* L., ranked as the most destructive biotic threat to rapeseed in Europe [27]. These beetles migrate within rapeseed fields to mate and lay eggs in autumn, shortly after sowing [79]; this is much earlier than the installation of the yellow sticky trap barrier, which would thus be powerless to control them, while the acetamiprid treatment applied in the same period would be able to reduce the beetle population already present in the plot. Larvae of this species burrow within stems and petioles, and start emerging from pupation as adults relatively late, in May [79]; this might explain why yield was lower even though seed pod damage incidence (associated with other pest species) was reduced in the sticky traps plot, and also why no adult beetles were observed during plant surveys. Of course, the lack of observation itself frames the cabbage stem flea beetle merely as a plausible explanation of the observed patterns; other potential explanations might include cumulative pest damage, damage to vegetative tissues not assessed in the study, differences in overwintering survival, plant vigor, spatial heterogeneity, or other biotic and abiotic factors.

### 4.4. Study Limitations

While considering these results, it should be once again stated that this was a small-scale pilot study, and all collected data should be interpreted with that in mind. Major limitations include (i) the fact that the study was carried out in a single site, with one plot for each pest control treatment (i.e., we only had one experimental unit per treatment, in which subsamples were collected), and (ii) the lack of a negative control plot (no sticky traps or insecticide) and of a plot with both pest control methods. Concerning the first limitation, while the experimental plots were adjacent (and thus the environmental conditions were presumably homogeneous across them), it would be impossible to separate spatial differences from treatment effects; the statistical analyses should thus be regarded as merely exploratory, and the results only potentially relevant for environmental conditions similar to those of the studied site. Concerning the second limitation, the lack of negative control prevents us from quantifying sticky-trap-associated reduction in pest pressure relative to the natural pest population, and the lack of a combined treatment prevents evaluation of whether yellow sticky traps could complement insecticide treatment or reduce the amount or frequency of insecticide application. In spite of this, our results allowed some interesting preliminary interpretations, with implications for the continuation of this line of research.

### 4.5. Practical Implications and Roadmap for Future Research

The present preliminary study provides preliminary evidence that yellow sticky traps may be a useful tool for the control of pollen beetles, weevils (including cabbage stem weevils) and associated pests in IPPM rapeseed agroecosystems, reducing damage incidence to seed pods while avoiding pollinator exposure to insecticides. Despite the higher proportion of damaged seed pods, however, the insecticide plot still had a higher yield, and a marginally improved contribution of pollinators to yield (in spite of a non-significant tendency for a higher pollination deficit). This implies that yellow sticky traps may need to be deployed alongside other complementary pest control methods for a more complete protection of the crop. To avoid any pollinator exposure to insecticides, future studies may investigate a combination of sticky traps with emerging pest-resistant cultivars [38], or with agroecological management for the enhancement of natural enemy communities [39,40,41]. Early installation (autumn, shortly after sowing) might also improve the efficacy of yellow sticky trap barriers, allowing them to intercept early invading pests such as the cabbage stem flea beetle, so this potential line of research should also be explored in the future. To improve result generalizability, future experiments should also use multiple independent field replicates and include untreated and combined treatments; monitoring should extend from autumn through harvest, so that pest species responsible for early-season, overwintering, and late-season damage can be identified.

## Figures and Tables

**Table 1:** Results of the Generalized Linear Mixed-Effects Models (GLMMs) and of the Generalized Linear Model (GLM—Model 2) testing the effect of pest control treatment (for all models), of pollination treatment and their interaction (for Models 3–4) on variables related to pest incidence and pollination contribution to yield.

Model Type	Dependent Variable	Fixed Effects	Df	Chi-Square	*p*
*Model 1*					
Poisson GLMM	Number of pest insects	Pest control treatment	1	190.08	<0.001
*Model 2*					
Binomial GLM	Pest-damaged seed pods (%)	Pest control treatment	1	27.30	<0.001
*Model 3*					
Gaussian GLMM	Yield per plant (g) ^a^	Pollination treatment	2	10.25	<0.01
		Pest control treatment	1	0.60	0.440
		Pollination × Pest	2	10.30	<0.01
*Model 4*					
Gaussian GLMM	Yield per flower (cg) ^b^	Pollination treatment	2	8.73	0.01
		Pest control treatment	1	2.74	0.09
		Pollination × Pest	2	7.06	0.03

^a^ Square-root transformed. ^b^ Log-transformed.

## Data Availability

The raw data supporting the conclusions of this article will be made available by the authors on request.

## Supplementary Materials

The following supporting information can be downloaded at https://www.mdpi.com/article/10.3390/insects17101035/s1: Figure S1: Experimental design for the pollination experiment and the collection of seeds in 1 × 1 plots; Figure S2: Abundance of the main pest species recorded on plants in the experimental plots during the rapeseed plant surveys (a) and abundance of the main pest species captured by yellow sticky traps (b); Figure S3: Abundance of honeybees (a) and other pollinator taxa (b) recorded visiting rapeseed flowers in the experimental plots. Vector art should be attributed to phylopic.org (Thomas Hegna: wild bee; Andy Wilson: all others); Figure S4: Box-and-whisker plot with medians, interquartile ranges, and overall ranges, showing the difference in yield (g/m^2^) between sticky trap treatment and the insecticide treatment, as a result of the exploratory statistical analysis.

## Abbreviations

The following abbreviations are used in this manuscript:

IPPM	Integrated Pest and Pollinator Management
GLMM	Generalized Linear Mixed-Effects Model
