# Supplementary material for: Advantages and Limitations of Yellow Sticky Traps as a Pest Control Method in Pollinator-Friendly Rapeseed Agroecosystems: A Pilot Study in Northern Italy
Source: Insects. 2026 Oct 9;17(10):1035. doi: 10.3390/insects17101035 (PMC13705304; doi:10.3390/insects17101035)
Supplement: Supplementary file 1 [file insects-17-01035-s001.zip › insects-4604575-supplementary.pdf]

SUPPLEMENTARY INFORMATION

Advantages and limitations of yellow sticky traps as a pest control method in pollinator-friendly rapeseed agroecosystems: A pilot study in Northern Italy

Francesco Lami[1], Olmo Scagliarini[2], Adele Bordoni[1,3], Lucia Lenzi[1], Serena Magagnoli[1], Marco Prandini[2], Giovanni Burgio[1], Fabio Sgolastra[1]

[1]Department of Agricultural and Food Sciences (DISTAL), University of Bologna, Viale Giuseppe Fanin 42, 40127 Bologna, Italy

[2]Centro Agricoltura Ambiente "G. Nicoli" S.r.l., Via Sant'Agata 835, 40014 Crevalcore (BO), Italy

[3]Department of Biology, University of Florence, Via Madonna del Piano 6, 50019 Sesto Fiorentino (FI), Italy

*Corresponding author: francesco.lami2@unibo.it

Running title: Pest control and pollination in rapeseed

Keywords: Pollination, Integrated Pest and Pollinator Management, Mass-flowering crops, *Brassicogethes aeneus*, *Ceutorhynchus obstrictus*, *Psylliodes chrysocephala*

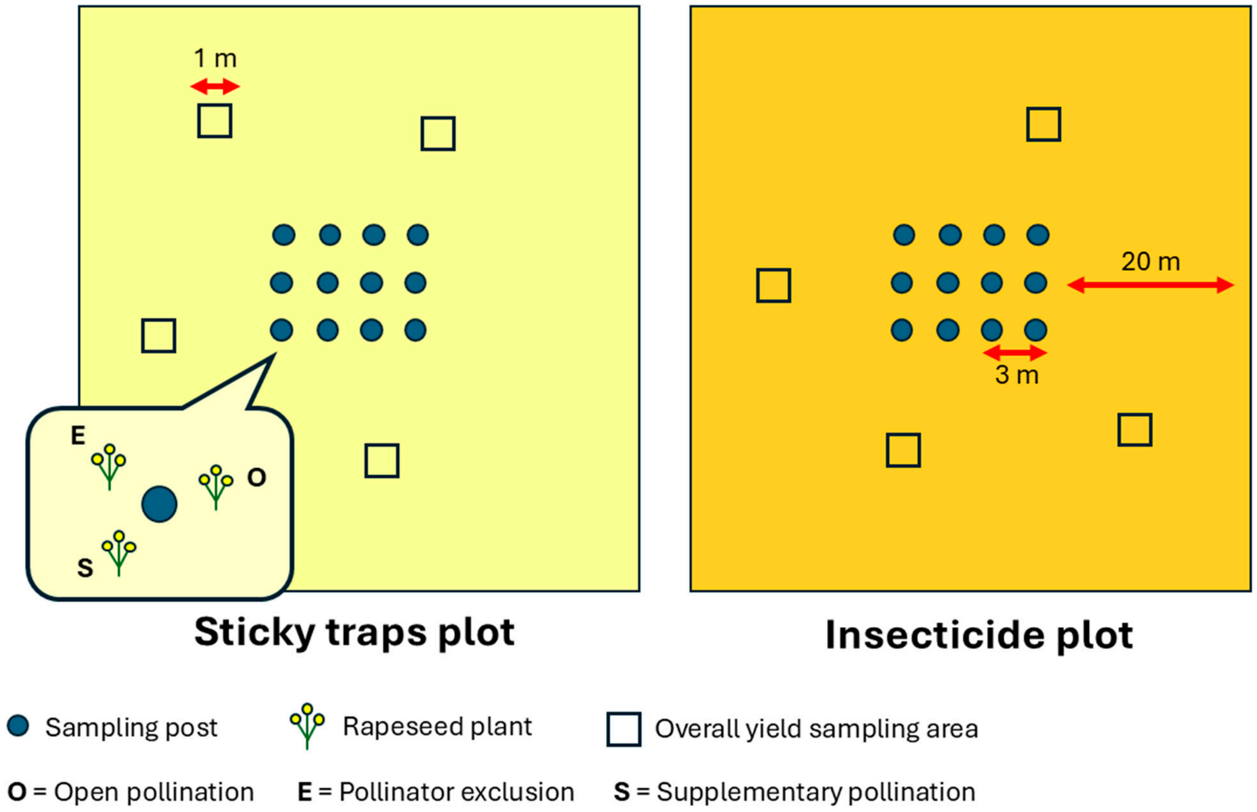

Figure S1. Experimental design for the pollination experiment and the collection of seeds in 1 × 1 plots.

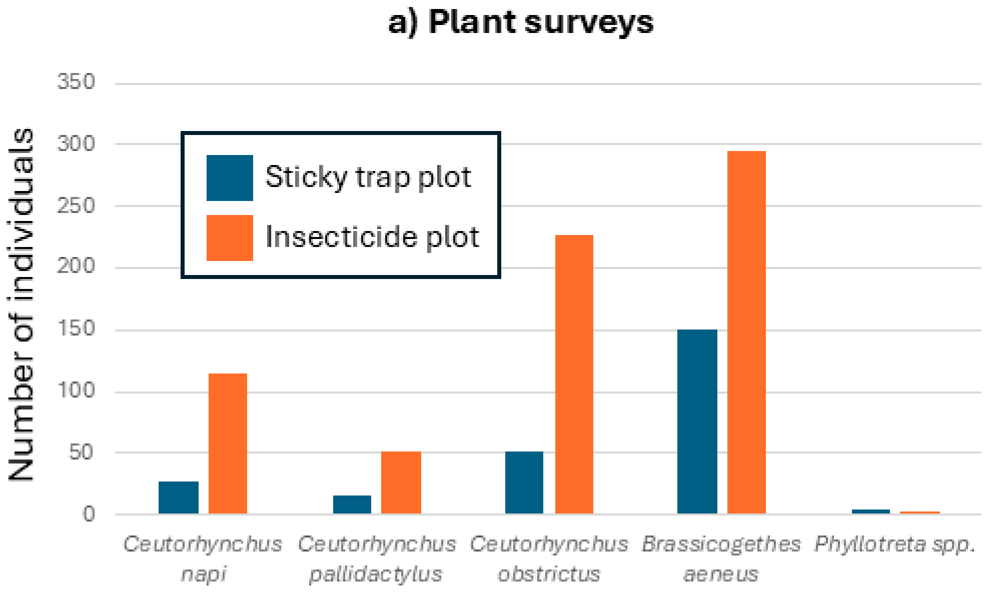

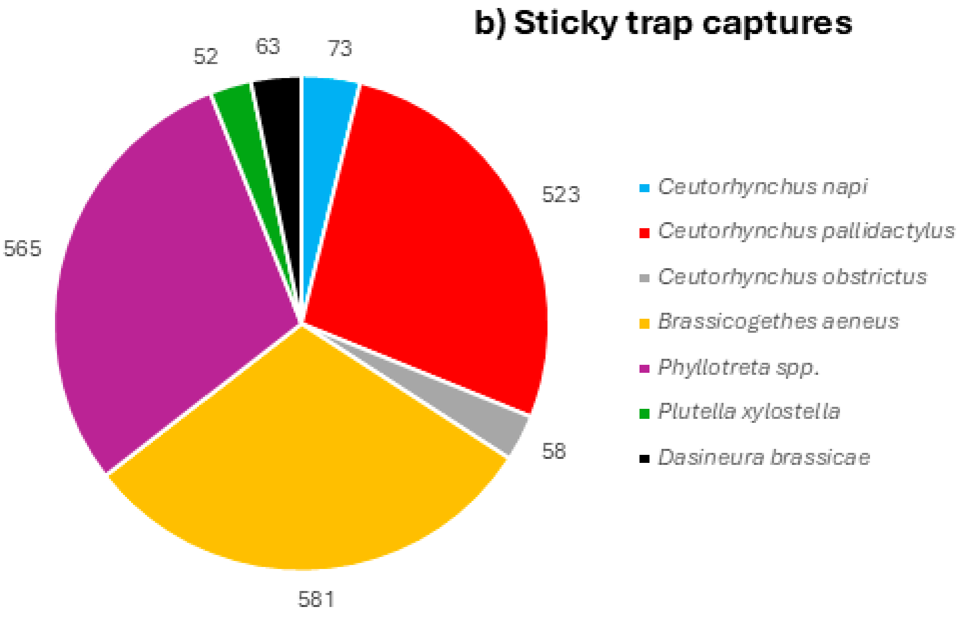

Figure S2. Abundance of the main pest species recorded on plants in the experimental plots during the rapeseed plant surveys (a) and abundance of the main pest species captured by yellow sticky traps (b).

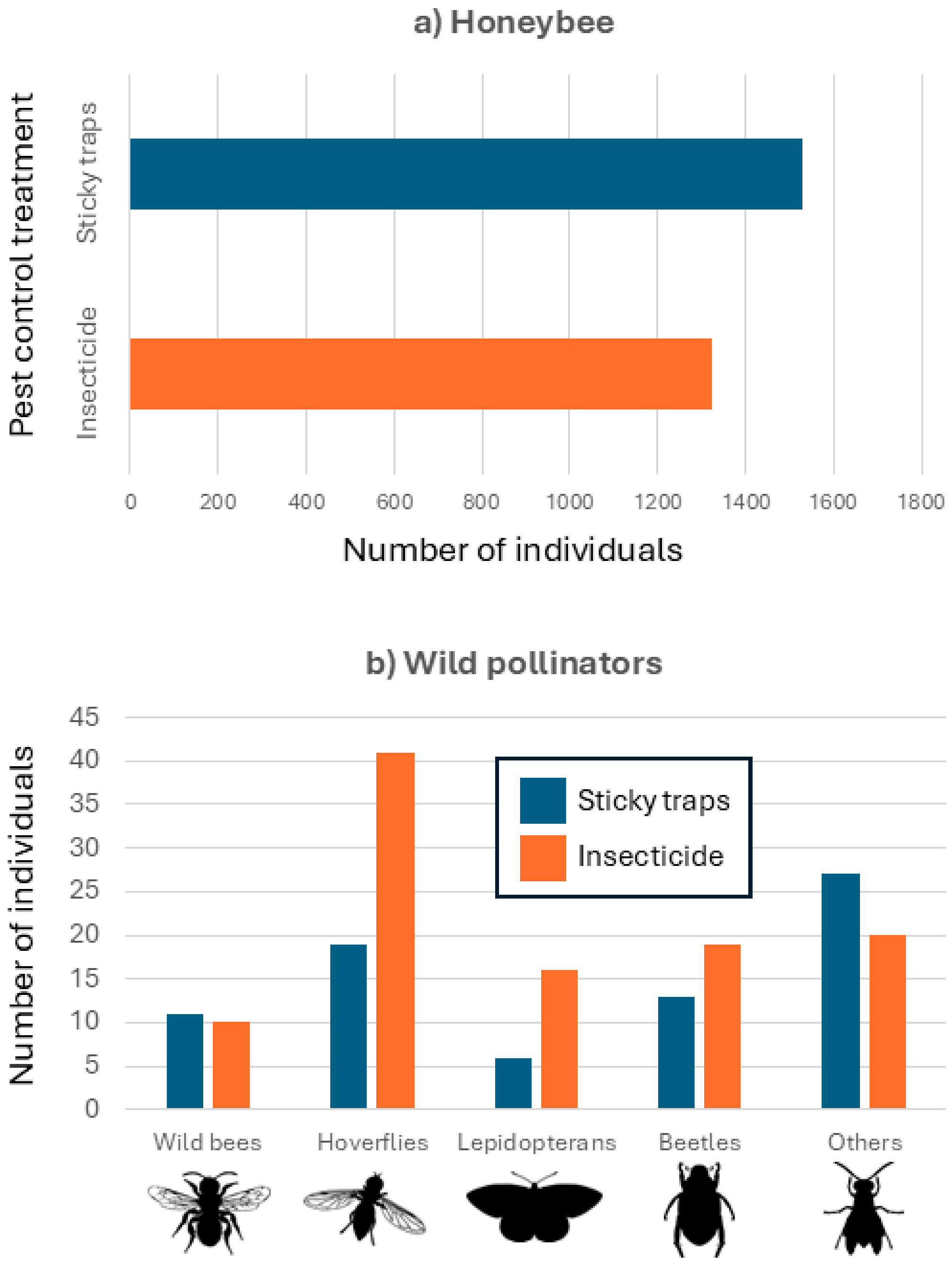

Figure S3. Abundance of honeybees (a) and other pollinator taxa (b) recorded visiting rapeseed flowers in the experimental plots. Vector art should be attributed to phylopic.org (Thomas Hegna: wild bee; Andy Wilson: all others).

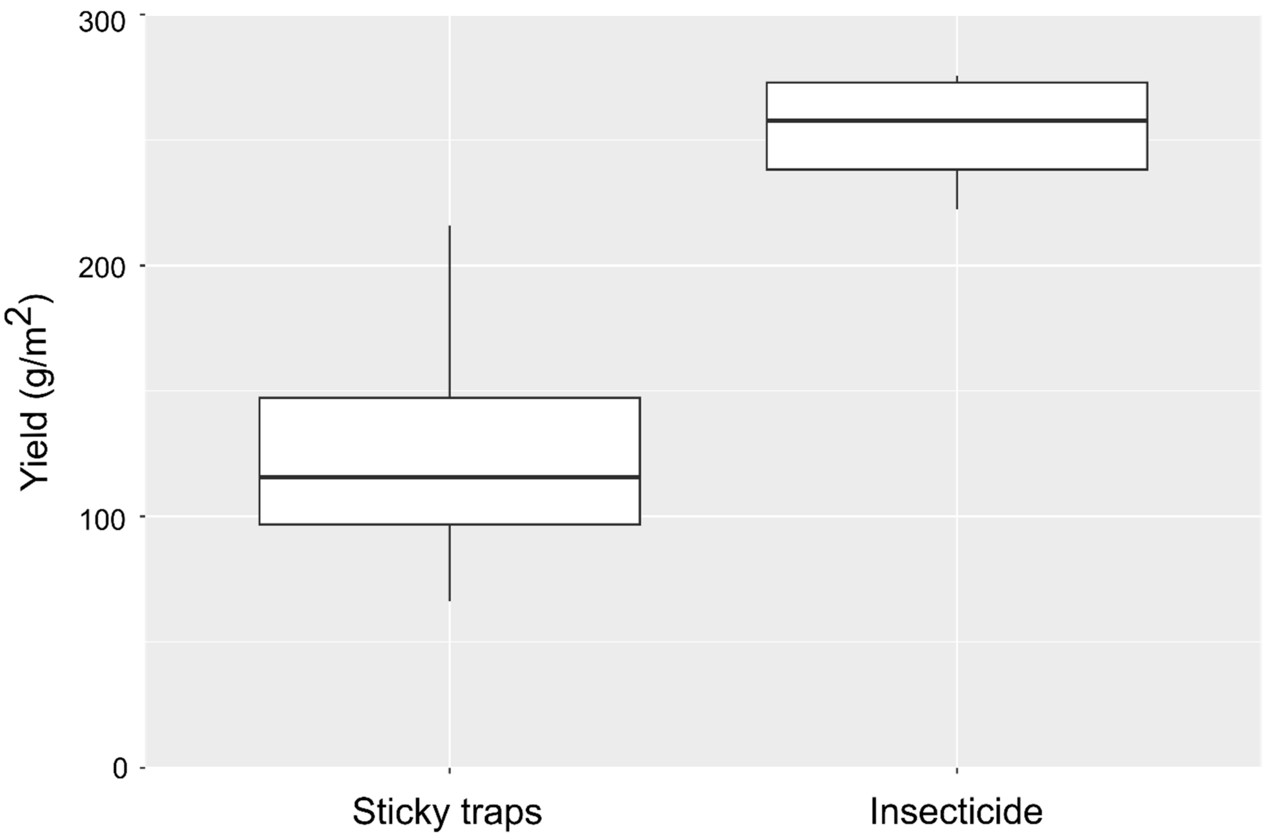

Figure S4. Box-and-whisker plot with medians, interquartile ranges, and overall ranges, showing the difference in yield (g/m^2) between sticky trap treatment and the insecticide treatment, as a result of the exploratory statistical analysis.
